# A Review of Recent Advances in the Treatment of Elderly and Poor Performance NSCLC

**DOI:** 10.3390/cancers10070236

**Published:** 2018-07-18

**Authors:** Juliet A. Carmichael, Daisy Wing-san Mak, Mary O’Brien

**Affiliations:** 1 Lung Unit, Royal Marsden Hospital, Downs Rd, Sutton, Surrey SM2 5PT, UK; jcarmichael2@nhs.net; 2 Queen Elizabeth Hospital, 30 Gascoigne Road, Yau Ma Tei, Hong Kong, China; daymakws@yahoo.com

**Keywords:** non-small cell lung cancer, Elderly, ECOG PS2, immunotherapy, EGFR TKIs, ALK inhibitors, ROS-1 rearrangement

## Abstract

Until recently, chemotherapy has remained the mainstay of treatment for the majority of patients with advanced non-small cell lung cancer (NSCLC). Excellent responses have been observed with immune-checkpoint inhibitors, and targeted treatments for those tumours with actionable mutations, resulting in a paradigm shift in the treatment approach for these patients. Elderly patients and those with poor performance status (PS), such as Eastern Cooperative Oncology Group (ECOG) 2, have historically been excluded from clinical trials due to poor outcomes. There is therefore a lack of data to define the optimal treatment strategy for these patients. Due to improved tolerability of novel therapies, inclusion of these patients in clinical trials has increased, and sub-group analyses have identified many treatments demonstrating potential activity. Here, we summarise key recent advances in the treatment of NSCLC, specifically evaluating their efficacy and tolerability in these patient cohorts.

## 1. Introduction

There have been major advances in the treatment of non-small cell lung cancer (NSCLC) in recent years. However, the optimal treatment of elderly patients, or those with poor performance status (PS) remains undefined, even though they represent a significant proportion of our patient population, with more than two-thirds of patients over 65 years of age [1]. The elderly and PS 2 patients are underrepresented, or often excluded from clinical trials. Here, we summarise key recent advances in NSCLC treatment in these sub-populations. 

## 2. Targeted Therapy in Elderly and/or PS2 Patients with Advanced NSCLC

One of the major advances in the treatment of stage IV NSCLC in recent decades was the recognition of “driver mutations” in a subset of patients (around 10% in non-Asian countries). It appears that these mutations or ‘oncogenes’, when found in the cancer, cause the cell to proliferate, thus blocking the function of this mutation results in tumour regression. The mutation is present for most if not all of the life of the patient and a continuing blockade in some form may be required—thus the term ‘oncogenic addiction’. Targeting these mutations results in disease control, improvement in quality of life and prolongation of progression-free survival when compared to chemotherapy. The most robust data are seen with tyrosine kinase inhibitors (TKIs) targeting sensitizing mutations in the epidermal growth factor receptor (EGFR) gene or targeting a fusion rearrangement of the anaplastic lymphoma kinase (ALK) gene, and these will thus be the focus of this review. 

Due to the relative ease of oral administration and favorable toxicity profile, trials of these agents have frequently included elderly patients and those with PS of 2. There are few trials specifically designed for these patients, and evidence of efficacy must be retrieved in the subgroup analysis of the large-scale studies, with key trials summarised in Table 1 and Table 2.

### 2.1. EGFR Mutations

Sensitizing mutations in the EGFR gene include exon 19 deletions and L858R point mutation in exon 21. These genetic changes are more prevalent among lung cancer patients with adenocarcinoma histology, of East Asian ethnicity, and who are never or light smokers [18,19]. The relationship between the presence of these somatic mutations with age is more controversial [20,21,22]. 

Four major trials on first generation TKIs (gefitinib and erlotinib), IPASS, IFUM, EURTAC, and OPTIMAL, included elderly patients of more than 65 years old (23–50% of total patients) and patients with PS2 (7–14% of total patients) [2,3,4,23]. In subgroup analyses, these trials showed that TKIs seem to confer greater benefits to elderly patients (>65 years), as shown by a lower hazard ratio (HR) in this subgroup. Although generally well tolerated, retrospective studies of gefitinib in elderly populations identified liver function derangement and skin rash as frequent adverse reactions [24,25]. Indeed, for erlotinib, it has been shown that elderly patients may experience more toxicity and require dose reduction [26,27]. A pharmacokinetic analysis of erlotinib in 53 patients demonstrated significantly greater plasma concentrations of erlotinib in elderly patients (>75 years), 1.5- to 2-fold higher than that observed in younger patients [28]. This was associated with greater rates of toxicity (100% versus 61%, all grades) and 83.3% (5/6) of those experiencing acute limiting toxicity were over 75 years [28]. This may be explained by altered pharmacokinetics in the elderly population, particularly reduced lean body mass, which was shown to be significantly lower in those >75 years, 36.6 versus 49.1 kg, *p* < 0.001 [28]. Therefore, appropriate dosing is key to limiting toxicity without compromising efficacy, and dedicated trials would be beneficial to establish optimal dosing in this cohort.

In patients with PS2, there is a general lack of data, although the OPTIMAL study failed to demonstrate any PFS benefit in this cohort with erlotinib. A phase III study of erlotinib versus placebo in patients with NSCLC unsuitable for chemotherapy included 564/670 (84%) patients with PS 2–3 [5]. PFS was significantly prolonged with erlotinib (Table 1), but median OS did not differ significantly between arms; 3.7 months with erlotinib (95% CI 3.2–4.2) versus 3.6 months (95% CI 3.2–3.9) in the placebo arm; unadjusted HR0.94, 95% CI 0.81–1.10, *p* = 0.46 [5]. However, significantly improved OS was observed in a sub-group of patients who developed first-cycle rash with erlotinib, a potential surrogate marker of efficacy [5]. Median OS in this cohort was 6.2 months (95% CI 4.8–7.2) with erlotinib, versus 4.1 months (95% CI 3.7–4.6) with placebo; HR 0.76 (95% CI 0.63–0.92, *p* = 0.0058) [5]. Rates of treatment-related adverse events (TRAE’s) were generally high, but were overall comparable between arms with 252/334 (75%) in the erlotinib arm versus 219/313 (70%) with placebo (grades 3–4; *p* = 0.12) [5]. This issue was also addressed in a phase II study, in which PS improvement rate was 79%, with 68% of patients with PS 3–4 achieving a PS < 1 with gefitinib [29]. 

Afatinib, a second-generation TKI, irreversibly blocks signalling from EGFR (EGFR/ErbB1), human epidermal growth factor receptor 2 (HER2/ErbB2) and ErbB4. In the subgroup analysis for elderly patients (>65 years) in LUX-Lung 3/6/7, afatinib improved progression free survival (PFS) but not overall survival (OS), except in the subset of elderly patients who harbour EGFR deletion 19 [30]. Grade 3/4 adverse reactions occurred in about half of these elderly patients, with diarrhoea and skin rash being the most common toxicities. Patients with PS2 were excluded from these studies [30]. TIMELY, a single arm phase II trial of afatinib (starting dose 40 mg) in patients with NSCLC unsuitable for chemotherapy with suspected or confirmed EGFR mutations, included 12/39 patients with ECOG PS2-3 [9,31]. Median PFS was 7.9 months (95% CI 4.6–10.2), with median OS of 15.5 months (95% CI 10.9–25.1) [9]. Toxicity rates, however, were greater than those observed in fitter populations, with 23/39 (59%) experiencing ≥1 grade 3 toxicity, most commonly diarrhea [9]. Whilst efficacy was greater in those with confirmed EGFR mutations, benefit was also seen in those with suspected EGFR mutations who would otherwise only be eligible for best supportive care [9].

Osimertinib is a third generation TKI-targeting double-mutant form of EGFR containing Thr790Met (T790M) mutation, which is the most common form of acquired resistance to first generation TKI. Both AURA3 and the pivotal FLAURA studies included patients with very advanced age (age range for osimertinib was 35–88 years and 25–85 in AURA3 and FLAURA, respectively) [10,11]. The benefit of osimertinib was maintained among all age subgroups. Toxicity rates were comparable across age subgroups, with diarrhoea and skin rash being the most common adverse reactions. Grade 3/4 toxicities were uncommon, with decreased appetite, prolonged QTc interval and diarrhoea being the most frequent severe events, appearing in 1–2% of patients. Patients with PS2 were excluded from these studies [11]. 

### 2.2. ALK Fusion Oncogene

Fusion of echinoderm microtubule-associated protein-like 4 (EML4) gene and ALK gene is found in approximately 5% of NSCLC [32]. It defines a distinct clinicopathologic subset of NSCLC, including patients with never or light smoking history, adenocarcinoma of signet ring or acinar histology, and younger age [33,34]. Similar frequencies of ALK gene rearrangements have been reported in East Asian and Western populations [35]. Key trial areas summarised in Table 2.

Crizotinib is a small molecule TKI of ALK, MET, and ROS1 kinase. It was shown to be superior to chemotherapy in the second and first-line setting in PROFILE 1007 and PROFILE 1014 trials, respectively [12,13]. Although the median age of patients on crizotinib was early 50’s, 16% of patients were >65 years in both studies, and <10% patients were PS2. The benefit of crizotinib in PFS was seen across all subgroups. As with first generation EGFR TKIs, the HR in elderly patients seems to signify more benefit than in their younger counterpart. Major grade 3/4 adverse reactions were elevated transaminases, gastrointestinal toxicities, and visual disturbance. Again, analysis of toxicities was not specific to elderly patients. 

Ceritinib is a second generation TKI, which is highly potent to ALK kinase receptor. Although it does not have MET inhibitor activity, it also targets insulin-like growth factor 1 receptor (IGF-1R), insulin receptor (InsR), and ROS1. In the two phase III studies, ASCEND-4 and ASCEND-5, comparing ceritinib to chemotherapy in first and second line, respectively, one-fifth of the study population were >65 year-old, and less than 10% of patients were PS2. Elderly patients derived similar benefits as those who were younger, but the evidence in PS2 is less clear—it was not reported in ASCEND-4, while those with PS1-2 in ASCEND-5 had an HR of 0.63 (95% CI 0.41–0.97), compared to 0.38 (95% CI 0.24–0.60) in those with PS 0 [14,15]. The most frequent G3/4 adverse reactions were raised transaminases, nausea and vomiting. Again, the analysis was not specific to elderly patients or those with poor PS. The ASCEND-8 trial compared the pharmacokinetics of the current dosing regimen of ceritinib (750 mg in fasted state) to lower doses of 450 mg or 600 mg, taken with a low-fat meal [36]. Interestingly, the lower dose of 450 mg had comparable systemic exposure to the standard dose of 750 mg, but with a more favorable GI toxicity profile, and thus lower rates of dose adjustments or interruptions [36]. The second part of this trial exploring efficacy is ongoing, and includes patients at extremes of age and PS 0–2 [37]. 

Alectinib is also a second generation TKI which inhibits ALK and RET. Following the remarkable response rate shown in the phase I/II studies [38], its superiority in terms of PFS and disease control in the central nervous system (CNS) over crizotinib was demonstrated in two phase III studies [16,17]. Patients included in both studies were in their 80’s and 90’s in J-ALEX and ALEX, respectively. In the ALEX study, elderly patients of >65 years had similar benefits to those younger, but when J-ALEX took the age cut-off at >75, there seemed to be no advantage of alectinib over crizotinib. Neither trial demonstrated benefit with alectinib over crizotinib in those with PS2, although numbers were small. There is no published data comparing tolerability within these sub-populations. 

### 2.3. ROS1- Translocation

More recently, ROS-1 rearrangements have been identified as oncogenic drivers, affecting 1–2% of NSCLC patients, with similar clinicopathologic characteristics to ALK-positive patients [39]. The ROS1 oncogene encodes a receptor tyrosine kinase, and chromosomal rearrangement results in constitutively activated ROS-1 fusion kinases which drive cell growth. There is significant homology between the kinase domains of ALK and ROS-1, and pre-clinical studies demonstrated activity of ALK inhibitors against ROS-1 mutated cell lines [39]. 

A phase I study of crizotinib in 50 patients with ROS-1 rearranged NSCLC demonstrated objective response rates (ORR) of 72% (95% CI 58–84), with a median PFS of 19.2 months (95% CI 14.4- not reached), and comparable tolerability to that reported in ALK-positive tumours [40]. Response here is significantly longer than that observed in ALK positive tumours, partly due to the natural history of ROS-1 mutated tumours, which tend to run a more indolent course. This trial included those up to 77 years, and one patient with PS 2, although specific outcomes in these cohorts were not reported. A retrospective analysis of 32 patients with ROS-1 mutated NSCLC demonstrated median PFS of 9.1 months in a heavily pre-treated population, and included patients up to 78 years, but again age-specific outcomes were not reported [41]. A case report of one 90-year old male with ROS-1 rearranged stage IIIA NSCLC treated with crizotinib, achieved a partial response, despite requiring a 50% dose reduction [42]. Several trials of crizotinib in this setting are ongoing, many of which include patients at extremes of age or PS2, with preliminary results consistent with previously reported efficacy, and subgroup analyses awaited [43,44,45,46]. A phase II study of ceritinib in 32 patients with previously-treated ROS-1 mutated NSCLC included patients up to 79 years and 4/32 patients with PS 2 [47]. Outcomes in these cohorts were not specifically reported, but median overall survival was 24 months (95% CI 5–43 months), and rates of TRAEs were generally low [47]. 

As frequently observed with targeted therapies, acquired resistance results in relapse, and several second and third generation ALK inhibitors have been developed to overcome this. Lorlatanib is a next generation ALK inhibitor which maintains activity across many known resistance mutations, and has good CNS penetrance [48]. A phase I trial has demonstrated efficacy in ROS 1 patients with PS 0–1, with 6/12 (50%) demonstrating an objective response [48], but its role in elderly patients or those with poor PS is yet to be determined.

Overall these studies highlight a potential role for ALK inhibitors in elderly or poor PS patients with ROS1- rearrangements. Dedicated trials in elderly or poor PS patients would be challenging, as it is a rare subtype, and more common in younger patients.

### 2.4. Targeted Therapy in Non-mutated NSCLC

Many trials have demonstrated benefit with targeted therapies in patients without actionable mutations, which is an attractive treatment approach for frail patients, and key trials are summarised in Table 3.

The first generation TKI, erlotinib, was previously considered a suitable option as second or third treatment for patients with advanced wild type EGFR (EGFRwt) NSCLC. This is based on the BR.21 study, which randomized patients who progressed on first or second line chemotherapy to erlotinib or placebo. These patients were unselected for EGFR mutation. The study showed a significantly improved OS (6.7 months vs. 4.7 months, HR 0.70; *p* < 0.001), in favour of erlotinib [49]. In this trial, around half of the patients were ≥60 years, and one-third had PS 2–3. A subsequent post-hoc analysis on BR.21 comparing survival between age groups, confirmed a sustained PFS and OS benefit in those over 70 years. Elderly patients, however, had significantly more overall and grade 3–4 toxicities (35% vs. 18%, *p* = 0.001), were more likely to discontinue treatment due to treatment-related toxicities (12% vs. 3%, *p* = 0.0001), and had lower relative dose intensity (64% vs. 82% received 90% of planned dose, *p* = 0.001) [26]. The authors concluded that elderly patients yielded similar benefits as younger patients, but suffered greater toxicities. The TITAN study compared erlotinib in the second line setting to standard chemotherapy (docetaxel or pemetrexed) in patients with poor prognosis, and included approximately 20% patients with PS 2, with age up to 80 years [50]. It demonstrated non-inferiority of erlotinib to chemotherapy in this cohort of patients, unselected for EGFR status, with less than 5% patients harboring an activating EGFR mutation.

The POLARSTAR study, a phase IV surveillance study on erlotinib in patients unselected for EGFR mutation status in second-line and beyond, focused on interstitial lung disease (ILD) and PFS. It demonstrated similarly low incidence of ILD in patients aged <75 years, 75–84 years, and ≥85 years; 4.2%, 5.1%, and 3.4%, respectively. The mortality rate from ILD was ≤1.7%. The PFS in these three groups ranged from 65 to 72 days [54]. Another retrospective study on 43 patients who failed first line chemotherapy yielded similar results: median PFS of 3 months (95% CI 0.4–28.4) and median OS of 8.4 months (95% CI, 0.7–43.6), with grade 3–4 toxicities observed in 37% [61].

However, chemotherapy is still the preferred agent for EGFRwt patients who progress after platinum-based doublet, especially in those who derived clinical benefit from first-line chemotherapy. This was confirmed in TAILOR, a randomised controlled trial of erlotinib vs. docetaxel in patients with EGFRwt NSCLC who had progressed following first line chemotherapy [51]. This included patients up to age 83, and 16/219 (7.3%) were PS2. In this trial of 219 patients, median PFS was superior with docetaxel (2.9 months versus 2.4 months, HR 0.71, 95% CI 0.53–0.95) as was median OS (8.2 months versus 5.4 months, HR 0.73, 95% CI 0.53–1.00). In the subgroup analysis, patients with PS 2 and those who progressed on platinum doublet did not derive greater benefit from docetaxel with respect to PFS or OS. Similar findings were demonstrated in the DELTA trial, whereby erlotinib was significantly inferior to docetaxel in EGFRwt NSCLC [52]. Furthermore, a meta-analysis of 1605 patients in 11 trials with EGFRwt NSCLC, showed that chemotherapy was superior to EGFR-TKIs, with HR for PFS of 1.84 (95% CI 1.35–2.52) and ORR 16.8 vs. 7.2% [62].

A phase II trial of first line erlotinib in chemotherapy-naïve elderly patients (≥70 years) with advanced NSCLC, unselected for EGFR, yielded a meaningful OS of 10.9 months (95% CI, 7.8–14.6 months). 8/82 (10%) patients enrolled were PS2. Acneiform rash and diarrhea affected more than two-thirds of patients, with grade 3–4 ILD in four patients, one of which resulted in treatment-related death [55]. Of note, those patients who developed treatment-related rash (63/80; 78.8%) had significantly longer time to progression and survival, with a median OS of 14.3 months in those with rash vs. 4.2 months in those without (17/80, 21.3%).

This finding has been demonstrated in many other trials [63,64,65], including the TOPICAL study, a randomized trial of erlotinib versus placebo in 770 patients who were deemed unsuitable for chemotherapy [5]. The incidence of EGFR mutation in this study was relatively low (28/390, 7%). The median age of patients was 77 years, and 564/670 (84.6%) were PS 2–3. Although the median OS did not differ between the two treatment groups, first cycle skin rash was found to be the only independent factor for survival, HR 0.24 (95% CI 0.16–0.35, *p* < 0.0001). Patients who developed first cycle skin rash on erlotinib (178/302, 59%) had significantly better OS vs. placebo (HR 0·76, 95% CI 0·63–0·92, *p* = 0·0058), while those who did not fared significantly worse (1·30, 1·05–1·61, *p* = 0·017). This benefit was observed across all subgroups, including those with PS3 (HR for OS 0.58, 95% CI 0.38–0.89, *p* = 0.012) and ≥75 years (HR for OS 0.77, 95% CI 0.61–0.97, *p* = 0.028). A positive correlation between rash severity and OS and PFS was also observed. Furthermore, the benefit of erlotinib was observed despite selecting a patient population with poor prognostic indicators, with the majority of patients being elderly with poor performance status and multiple co-morbidities. This therefore represents a viable treatment option in this cohort, and the development of first cycle rash provides a valuable clinical biomarker of efficacy. The biological rationale for this observation is unclear, but may reflect altered pharmacokinetics and therefore drug exposure, or may be a surrogate marker of the immune response against tumour [5].

Chen et al conducted a randomized phase II study of erlotinib vs. vinorelbine in chemotherapy-naive, EGFR unselected Taiwanese patients ≥70 years [53]. 26/113 (23%) patients were PS 2–3. The response rate (22.8% vs. 8.9%, *p* = 0.04) and median PFS (4.6 months vs. 2.5 months, *p* = 0.03) were significantly better with erlotinib, although median OS was similar (11.7 months for erlotinib vs. 9.3 months for vinorelbine, *p* = 0.70). In the subgroup analysis, improved survival was observed in patients with EGFR mutations, irrespective of the treatment they received. 

SATURN, a randomised phase III trial of erlotinib maintenance versus placebo after first-line platinum-doublet chemotherapy in unresectable NSCLC, demonstrated a survival benefit in all comers with maintenance erlotinib [57]. 889 patients were randomised 1:1 after completion of chemotherapy, with a median age of 60 (30–83) and all patients were ECOG PS 0–1. 388/889 (43.6%) patients were EFGRwt, whilst for 443/889 (49.8%) patients, EGFR mutation status was ‘indeterminate’ or ‘missing’. PFS and OS were significantly prolonged with erlotinib, irrespective of EGFR mutation status, with a HR for OS of 0.77 (95% CI 0.61–0.97; *p* = 0.0243) and a HR for PFS of 0.78 (0.63–0.96; *p* = 0.0185) in EGFRwt tumours.

Less data is available to support the use of gefitinib in elderly patients or those with poor PS who are EGFRwt or unselected. In IFCT-0301, a phase 2 study by Des Guetz, patients unselected for EGFR mutation status were randomized to first-line gefitinib, gemcitabine or docetaxel [56]. In this study, more than half of the patients were ≥70 years, and all patients were PS 2–3. Toxicities were mild, although they were more severe in the docetaxel group, and diarrhoea was more common with gefitinib. PFS was 1.4 months (95% CI, 1.1–1.9) for younger (<70 years) compared to 2.3 months (95% CI, 2.1–2.9) for elderly patients (≥70 years), while OS was 2.0 months (95% CI, 1.5–2.4) and 3.7 months (95% CI, 2.4–4.8), respectively. Subgroup analysis found that age, but not choice of treatment, was the main prognostic factor for PFS (adjusted HR 0.57, 95% CI, 0.38–0.85) for the elderly compared to patients aged <70 years (*p* = 0.004). INTEREST, a randomised phase III trial, showed that gefitinib was non-inferior to docetaxel as second-line treatment in unselected NSCLC [58]. In contrast, the CTONG0806 trial demonstrated significant improvement in PFS (4.8 vs. 1.6 months; HR 0.54, 95%CI 0.40–0.75; *p* < 0.001), and a trend for better OS (12.4 vs. 9.6 months) with pemetrexed over gefitinib in second-line treatment of EGFRwt NSCLC [59].

There is limited data supporting the safety and efficacy of afatinib in patients with EGFRwt NSCLC. A phase II single-arm study of afatinib in Korean patients with EGFRwt advanced NSCLC who had progressed despite two lines of chemotherapy, but no prior anti-EGFR treatment, recruited 42 patients [60]. No complete or partial responses were observed, and only 24% achieved stable disease for more than 6 weeks. TIMELY, a phase II trial of first-line afatinib in patients unsuitable for chemotherapy with suspected or confirmed EGFR-mutated NSCLC recruited 39 patients. Median age was 72 years (range 36–90 years), of which 19/39 (48.7%) had suspected but unconfirmed EGFR mutations, and an overall 6-month PFS rate of 58% was shown. Although relatively high rates of TRAE’s were observed (23/39, 59% experienced grade 3–4 toxicities), efficacy was demonstrated in those with suspected but unconfirmed EGFR mutations, with median OS of 10.9 months (95% CI 3.9–21); a cohort of patients who would otherwise only be eligible for best supportive care (BSC). The full results of the trial are awaited, including relative efficacy in elderly patients and those with poor PS [9]. 

In summary, the best therapeutic options for elderly or poor PS patients are still best dictated by driver mutations. However, these studies suggest that EGFR-TKIs are valid options in patients who are unselected or even negative for EGFR mutation status. Only 29% of patients with newly-diagnosed NSCLC proceed to chemotherapy in the UK [5]. The potential benefit of targeted therapies is therefore of particular importance in patients deemed unfit for platinum-based chemotherapy, in whom there is an unmet need for therapeutic options. Several trials have highlighted the importance of first-cycle rash as a predictive biomarker of response, and this could be used at an early stage to identify patients likely to benefit from EGFR-TKIs.

## 3. Chemotherapy in Elderly and/or PS2 Patients with Advanced NSCLC

For patients without actionable mutations, platinum-based chemotherapy remains the standard of care in NSCLC. Early trials demonstrated poor survival, high rates of TRAE’s and treatment-related deaths in patients with ECOG PS2 [66,67], and they were therefore excluded from many subsequent trials. The optimal treatment regimen for elderly patients or those with poor PS remains undefined, and key trials are summarised in Table 4. Current ASCO/ESMO guidelines recommend platinum doublet chemotherapy for patients, irrespective of age, and consideration of attenuated-dose doublet chemotherapy or monotherapy in those with poor performance status [68,69].

### 3.1. Two versus One Drug

The landmark ELVIS trial was the first prospective trial to specifically evaluate elderly patients with NSCLC [75]. It demonstrated a survival benefit of vinorelbine against best supportive care (BSC) of 28 weeks versus 21 weeks, with associated improvement in quality of life [75].

Multiple trials have since explored the role of various different combination regimens [76,77,78,79,80]. Pemetrexed is an anti-folate agent which has demonstrated efficacy with a favorable toxicity profile, and may therefore represent a good treatment option for elderly patients [81,82]. A meta-analysis of 3 randomised controlled trials (RCTs) conducted in East Asia demonstrated increased ORR of 32.8 vs. 7.5%, and non-significantly prolonged overall survival with carboplatin/pemetrexed over other platinum-based doublet combinations [83]. Importantly, the incidence of grade 3/4 TRAEs was lower with carboplatin/pemetrexed. 

Pemetrexed has also demonstrated efficacy as monotherapy. A sub-group analysis of a randomised phase III trial of pemetrexed versus docetaxel in the second-line setting demonstrated non-inferior overall survival benefit with pemetrexed in elderly patients, 9.5 months versus 7.7 months (HR, 0.86; 95%CI, 0.53–1.42), as well as enhanced tolerability [84]. Dedicated phase II trials of pemetrexed monotherapy in the elderly have also demonstrated efficacy [85,86,87], but this is not yet licensed in this setting.

The IFCT-0501 trial compared carboplatin/paclitaxel to monotherapy with either vinorelbine or gemcitabine in patients over 70 years [78]. Whilst more toxicity was observed in the combination arm, median overall survival was prolonged to 10.3 months, compared to 6.2 months with monotherapy (HR 0.64, 95% CI 0.52–0.78, *p* < 0.0001) [78]. In fact, the subgroup analysis also demonstrated improved OS in those over 80 years, with HR of 0.53 (95% CI 0.36–0.80) [78]. Thus, until recently, two drugs in the elderly were considered better than one.

Two parallel phase 3 trials have been carried out to further explore combination therapy in the elderly (>70 years), MILES 3 and 4 [70,88,89]. MILES-3 compared cisplatin/gemcitabine against gemcitabine alone in patients with NSCLC. MILES-4 compared gemcitabine/cisplatin against gemcitabine and cisplatin/pemetrexed against pemetrexed in patients with non-squamous NSCLC [70,88]. Patients with PS ≥2 were excluded. The trials have been closed early due to slow accrual, and the results have been pooled (531 patients) for combined analysis. The interim results recently reported demonstrate significant prolongation of PFS (4.6 vs. 3 months, HR 0.76, 95% CI: 0.63–0.92, *p* = 0.005) and an increased response rate of 15.5% vs. 8.5% (*p* = 0.02) with the addition of cisplatin, without significant change in median OS, 9.6 versus 7.5 months (HR 0.86, 95% CI: 0.70–1.04, *p* = 0.14) [70,88]. Of note, there was significantly more haematological toxicity and fatigue, but no significant change in QOL with cisplatin [90]. Thus, once again data are suggesting and supporting single age chemotherapy for the elderly on the basis of acceptable toxicity.

The IFCT-0501 trial included 123/450 (30%) patients with PS2, and the survival benefit of doublet chemotherapy was preserved in these patients, with HR for OS of 0.63 (95% CI 0.43–0.91) in PS2, versus 0.63 (95% CI 0.49–0.81) in PS 0–1 [78]. Subgroup analysis of another phase III trial, showed significant prolongation of overall survival in 50 ECOG PS 2 patients treated with carboplatin/paclitaxel versus paclitaxel alone (4.7 versus 2.4 months, *p* = 0.016) [91]. 

ECOG 1599, a randomised phase II trial of dose attenuated carboplatin/paclitaxel versus cisplatin/gemcitabine, was the first dedicated trial of NSCLC in patients with ECOG PS2 reported in 2007 [92]. One-hundred-and-three patients were recruited, and despite dose reduction, survival was significantly greater than previously documented, 6.2 months (carboplatin/paclitaxel) versus 6.9 months (cisplatin/gemcitabine). Furthermore, TRAEs occurred at a similar rate to that previously observed in the ECOG PS 0–1 population. A similar trend was observed in the CAPPA-2 trial, a dedicated phase III study of first line gemcitabine/cisplatin versus gemcitabine in 57 patients with ECOG PS 2, with OS 5.9 versus 3 months, *p* = 0.039, in favour of the combination [93]. 

Improved outcomes have been observed with pemetrexed regimens, which are more tolerable in frail patients. A phase III trial of first line pemetrexed versus carboplatin/pemetrexed (4 cycles) in 205 patients with ECOG PS 2 demonstrated a significant survival benefit with combination therapy, with a median OS of 9.3 months versus 5.3 months (HR, 0.62; 95% CI, 0.46–0.83; *p* = 0.001) and objective RR of 24% versus 10.5% [94]. Although haematological toxicity was higher with the combination, grade 3–4 toxicity rates were otherwise low, although there were four treatment-related deaths in the combination arm. A dedicated phase II trial of first line pemetrexed as monotherapy in 28 patients with non-squamous NSCLC demonstrated reasonable efficacy, with a median overall survival of 9.5 months (95% CI 3.3–12.5) [95]. Again, low rates of grade 3–4 TRAEs were observed, predominantly myelosuppression [95]. The conflicting survival data here are likely to be a reflection of the heterogeneity within this cohort, but pemetrexed monotherapy represents a reasonable treatment option for patients unfit for doublet chemotherapy.

### 3.2. New Agents

A phase III trial of carboplatin with weekly albumin-bound paclitaxel (nab-paclitaxel) versus carboplatin with three-weekly solvent-based paclitaxel (sb-paclitaxel) of 1052 patients with untreated, advanced NSCLC demonstrated significantly improved ORR of 33% vs. 25% (response rate ratio, 1.313; 95% CI 1.082–1.593; *p* = 0.005) and an improved toxicity profile with nab-paclitaxel [71]. The trial predominantly included PS 0–1 patients, with only 5/1052 (<1%) PS2. Whilst modest improvements in PFS and OS were observed, this was not sufficient to reach statistical significance [71]. Interestingly, subgroup analysis revealed significantly increased OS with nab-paclitaxel in those ≥70 years, 19.9 vs. 10.4 months with sb-paclitaxel; *p* = 0.009 [71]. 

This data prompted a dedicated phase IV randomized trial of nab-paclitaxel in the elderly population, ABOUND 70+ [72]. Patients ≥70 years with locally advanced/metastatic NSCLC were randomised 1:1 to carboplatin plus nab-paclitaxel, administered either weekly without a break (arm A), or weekly for 3 weeks followed by a 1 week treatment break, in a 28 day cycle (arm B). Full results are awaited. Median cumulative dose was higher in arm B (1287.5 vs. 875 mg/m^2^) due to fewer dose reductions, delays or missed doses, but the toxicity profile was similar across treatment arms [72]. ORR was greater in arm B, 40.3% vs. 23.9% (*p* = 0.037), with significantly prolonged PFS of 7.0 vs. 3.9 months (HR 0.49; 95% CI, 0.30–0.79; *p* = 0.003), although there was no significant difference in OS (16.2 vs. 15.2 months, HR 0.76; 95% CI, 0.46–1.26; *p* = 0.292). This trial demonstrated greater OS figures with nab-paclitaxel than previous trials.

ABOUND PS2 is a phase II trial of induction carboplatin/nab-paclitaxel (4 cycles) followed by nab-paclitaxel monotherapy as first line treatment of NSCLC in 40 patients with ECOG PS 2 [73,96,97]. Whilst 9/40 patients (22.5%) discontinued due to TRAE’s during the induction phase, 16/40 (40%) went on to receive nab-paclitaxel monotherapy, with 4/40 patients (10%) continuing on therapy beyond 11 cycles at time of data cut off. Median PFS was 4.4 months (95% CI 3.2–5.7) with median OS of 8.6 months (95% CI 5.1–13.2); an objective improvement in quality of life was observed. 

The other new group of drugs inhibit the vascular endothelial growth factor (VEGF) pathway, including small molecule inhibitors (nintedanib) and antibodies (bevacuzumab and ramicurimab). In general, there is caution with the use of these agents in the elderly and PS 2 and, therefore, neither group were included in the registration trials [1]. 

### 3.3. Maintenance Chemotherapy

Maintenance chemotherapy in general in NSCLC has been another improvement in therapy. The role of maintenance therapy in these subpopulations remains unproven, but a recent meta-analysis of five RCTs suggested a survival benefit with single-agent maintenance therapy in elderly patients with NSCLC [98]. The PARAMOUNT study demonstrated a survival benefit with pemetrexed maintenance therapy following platinum-based induction therapy, and included elderly patients but excluded those with PS ≥2 [74,99]. Those ≥70 years (92/539 patients; 17%) demonstrated a significantly prolonged PFS with maintenance pemetrexed, with a HR for PFS of 0·35 (95% CI 0·20–0·63), but failed to demonstrate an overall survival benefit [74,99]. The rate of adverse events was no greater than in the overall study population [74,99]. A single-arm phase II trial of carboplatin/pemetrexed followed by pemetrexed in 34 elderly patients with non-squamous NSCLC demonstrated good survival outcomes, comparable to those observed in the overall population, with a median PFS of 5.7 months and median OS of 20.5 months, although patient numbers were low [100]. A randomised phase III trial (JCOG-1210) of carboplatin/pemetrexed followed by pemetrexed maintenance versus docetaxel specifically in the elderly population is on-going [101].

## 4. Immunotherapy in Elderly and/or PS2 Patients with Advanced NSCLC

The introduction of immune checkpoint inhibitors targeting the programmed-death 1/programmed-death ligand 1 (PD-1/PD-L1) and/or cytotoxic T-lymphocyte–associated antigen 4 (CTLA-4) pathways has improved the treatment of advanced NSCLC in recent years, with several RCTs demonstrating survival advantage against or in combination with chemotherapy, in first and subsequent lines of therapy [102,103,104,105,106,107,108,109,110]. Furthermore, recent data also suggests a role in stage III following chemoradiotherapy [108], and in the neo-adjuvant setting [111]. Subgroup analyses of many of the key RCTs have demonstrated survival benefit across all age groups, without significant increase in toxicity; however, patients with PS2 have been excluded from the majority of trials. Key trials are summarised in Table 5 and Table 6.

### 4.1. Single-Agent Immunotherapy

Checkmate 153 (on-going) is a phase III/IV trial investigating optimal duration of Nivolumab treatment, whereby previously treated patients were randomised to receive Nivolumab for 1 year, or until progression/unacceptable toxicity/withdrawal of consent [109]. 544/1375 (40%) of those treated were ≥70 years, 123/1375 (9%) patients were PS2, and many patients were heavily pre-treated. Preliminary data has shown similar survival across all age groups, with estimated median OS of 9.4 months (95% CI 8.3–10.9) in those <70 years, and 10.3 months (95% CI 8.3–11.6) in those ≥70 years. As expected, the estimated median OS was lower for PS2 patients at 3.9 months (95% CI 3.1–6.3), compared to 10.5 (95% CI 9.3–11.4) in PS 0–1. Higher rates of death and requests to discontinue treatment resulted in a shorter duration of therapy in PS2 patients (median 1.4 months versus 3.5 months).

Importantly, rates of TRAEs were comparable across age groups, with 90/830 (11%) versus 73/544 (13%) in those ≥70 years (grade 3–4). Rate of discontinuation of treatment due to toxicity was low across all subgroups (3% <70 years, 4% ≥70 years, 3% PS2). Frequency of TRAEs was the same in the ECOG PS 2 subgroup, 13/123 (11%) in PS 2 versus 146/1230 (12%) in PS 0–1 (grades 3–4). In all subgroups, including PS2, there was a statistically significant improvement in symptom burden and health-related quality of life (QoL) at most time points. 

Checkmate 171 (ongoing) is a single arm phase 2 trial of Nivolumab in pre-treated patients with squamous NSCLC, with a planned exploratory analysis of those aged ≥70 years and PS2 [110]. 279/809 patients (34.5%) were ≥70 years, and 98/809 (12%) were PS2. Preliminary data has demonstrated similar estimated median OS in the elderly and overall study population, at 11.2 months (95% CI 7.6-not available) in ≥70 years versus 9.9 months overall (95% CI 8.7–13.1), again with reduced median OS in PS2 patients at 5.4 months (95% CI 3.9–8.3). Partial response rate at week 9 was 14% in the overall study population, 14% in those ≥70 years, and 11% in PS2 patients. Rates of TRAEs were comparable, with 95/809 (12%) overall, 38/279 (14%) in ≥70 years and 6/98 (6%) in PS2 patients (Grades 3–4). Again, the median duration of treatment was lower in those with ECOG PS 2 (95% CI 1.8 months, 1.2–2.8), compared to the overall study population (95% CI 4.4 months, 3.9–4.9). 

### 4.2. Combination Immunotherapy

There has been recent interest in the role of combination immunotherapy. Checkmate 227 is an open-label phase III trial of upfront Nivolumab (+chemotherapy in those with PDL-1 < 1%) versus Nivolumab plus Ipilimumab versus chemotherapy alone (1:1:1) in stage IV or recurrent NSCLC with high mutational burden [104]. Detailed subgroup analysis of TRAEs and efficacy specifically across age groups has not yet been reported. However, as summarised in Table 5, HR for PFS with combination immunotherapy versus chemotherapy was 0.62 (95% CI 0.40–0.97) in those 65 years or above, which although less impressive than for those under 65 years, still demonstrates significant improved activity over chemotherapy alone. Although the HR for those 75 years or above is not significant, the confidence interval is wide due to low patient numbers, making this difficult to interpret.

Keynote-189 is a phase III trial of upfront platinum/pemetrexed with pemetrexed maintenance, with or without Pembrolizumb, in metastatic non-squamous NSCLC [103]. 304/616 (49.4%) patients were aged 65 or above. The first interim analysis demonstrated OS benefit of Pembrolizumab combination therapy in all subgroups, with HR for OS of 0.64 (95% CI 0.43–0.95) in ≥65 years, although this was less significant than the <65 years cohort (see Table 5). The HR for PFS was, however, not significant in ≥65 years, with HR 0.75 (95% CI 0.55–1.02).

The inclusion of elderly patients in these trials has been greater than in the historic chemotherapy trials, but the numbers remain too small for adequately powered subgroup analyses. Overall, these studies have shown that immunotherapy is efficacious, without significantly greater toxicity than in younger patients, or with alternatives such as chemotherapy, and represents an important therapeutic strategy for these patients, who are PS 0 or 1 and who could tolerate the potential toxicities. The preliminary data from checkmate 171/153 demonstrates that immunotherapy is safe and well tolerated in PS2 patients, with reasonable efficacy, although survival outcomes remain relatively poor. There is a need for further dedicated trials in these cohorts to further evaluate the role of immunotherapy in these patients. Of note, two dedicated phase 2 trials of immunotherapy in ECOG PS2 patients are on-going, Pembrolizumab in previously treated NSCLC (PEPS-2) [112], and first line Durvalaumab (MEDI4736) [113].

## 5. Conclusions

There have been significant recent advances in the treatment of NSCLC, and the field is rapidly evolving. However, the management of elderly patients and those with poor PS remains challenging, despite representing a significant proportion of our caseload. Due to historical lack of inclusion in clinical trials, there remains a paucity of data to guide the optimal management of these patients, and much of the guidance is based on retrospective subgroup analyses alone. 

Standard chemotherapy for the elderly in trials is still oscillating between single agent and combinations. For PS 2 patients, combinations appear to be better, even at attenuated doses. There are no new drugs to be added to the standard list for now. Targeted therapy appears applicable to all patients now in view of the excellent tolerability, and may play a role in wild-type NSCLC for some patients. Immunotherapy appears to be tolerable and efficacious in the elderly population, and will change the natural history of this disease. The elderly have been included in the main practice changing trials and should continue to be included.

Despite these advances, outcomes for patients with poor performance status remain sub-optimal. Whilst immunotherapy appears to be safe and tolerable in this cohort, survival remains poor, suggesting that the impact on the natural history is less dramatic. As this is a highly heterogeneous population, in whom poor PS may reflect cancer-related morbidity or other co-morbidities, small subgroup analyses are insufficient to generate meaningful outcomes. Generating more robust information on the discriminating role of PDL1 expression or tumour mutation burden should be the basis of randomisation of trials which are now dedicated to PS 2 patients.

## Figures and Tables

**Table 1:** Sub-group analyses of elderly patients in phase II/III trials of first line EGFR TKI’s in patients with advanced NSCLC.

Trial	Age (Years)	PS	Regimen	Patients (n)	Median PFS, Months (95% CI)	HR for PFS (95% CI)
IPASS [2]	<65	0–2	Gefitinib Chemo	899	NR	0.81 (0.70–0.95)
	≥65	0–2	Gefitinib Chemo	318	NR	0.58 (0.45–0.76)
OPTIMAL [3]	<65	0–2	Erlotinib Chemo	116	NR	0.19 (0.11–0.31)
	≥65	0–2	Erlotinib Chemo	38	NR	0.17 (0.07–0.43)
EURTAC [4]	<65	0–2	Erlotinib Chemo	85	NR	0.44 (0.25–0.75)
	≥65	0–2	Erlotinib Chemo	88	NR	0.28 (0.16–0.51)
TOPICAL [5]	77 (72–82)	0–3	Erlotinib Placebo	350 320	2.8 (2.6–3) 2.6 (2.4–2.9)	0.83 (0.71–0.97) *p* = 0.019
LUX-LUNG 3 [6]	<65	0–1	Afatinib Chemo	211	NR	0.53 (0.36–0.76)
	≥65	0–1	Afatinib Chemo	134	NR	0.64 (0.39–1.03)
LUX-LUNG 6 [7]	<65	0–1	Afatinib Chemo	278	NR	0.30 (0.21–0.43)
	≥65	0–1	Afatinib Chemo	86	NR	0.16 (0.07–0.40)
LUX-LUNG 7 [8]	<65	0–1	Afatinib Gefitinib	177	NR	0.68 (0.48–0.97)
	≥65	0–1	Afatinib Gefitinib	142	NR	0.85 (0.59–1.22)
TIMELY (*Phase II*, *single arm*) [9]	36–90	0–3	Afatinib	39	7.9 (4.6–10.2)	NR
AURA3 [10]	<65	0–1	Osimertinib Chemo	242	NR	0.38 (0.28–0.54)
	≥65	0–1	Osimertinib Chemo	177	NR	0.34 (0.23–0.50)
FLAURA [11]	<65	0–1	Osimertinib Gefitinib or Erlotinib	298	NR	0.44 (0.33–0.58)
	≥65	0–1	Osimertinib Gefitinib or Erlotinib	258	NR	0.49 (0.35–0.67)

**Abbreviations**: PS—performance status; HR—hazard ratio; PFS—progression free survival; CI—confidence interval; NR—not reported; Chemo—Chemotherapy.

**Table 2:** Sub-group analyses of elderly patients in phase III trials of ALK inhibitors in patients with advanced NSCLC.

Trial	Age (years)	Tx Line	PS	Regimen	Patients (n)	Median PFS, Months (95% CI)	HR for PFS (95% CI)
PROFILE 1007 [12]	<65	2	0–2	Crizotinib Chemo	297	NR	0.49 (0.37–0.65)
	≥65	2	0–2	Crizotinib Chemo	50	NR	0.54 (0.27–1.08)
PROFILE 1014 [13]	<65	1	0–2	Crizotinib Chemo	288	NR	0.51 (0.38–0.68)
	≥65	1	0–2	Crizotinib Chemo	55	NR	0.37 (0.17–0.77)
ASCEND-4 [14]	<65	1	0–2	Ceritinib Chemo	295	NR	0.58 (0.42–0.80)
	≥65	1	0–2	Ceritinib Chemo	81	NR	0.45 (0.24–0.86)
ASCEND-5 [15]	<65	2	0–2	Ceritinib Chemo	178	NR	0.53 (0.37–0.77)
	≥65	2	0–2	Ceritinib Chemo	53	NR	0.26 (0.12–0.58)
J-ALEX [16]	<65	1	0–2	Alectinib Crizotinib	185	NR	0.34 (0.21–0.56)
	≥65	1	0–2	Alectinib Crizotinib	22	NR	0.28 (0.06–1.19)
ALEX [17]	<65	1	0–2	Alectinib Crizotinib	233	NR	0.48 (0.34–0.70)
	≥65	1	0–2	Alectinib Crizotinib	70	NR	0.45 (0.24–0.87)

**Abbreviations**: PS—performance status; Tx line—treatment line; HR—hazard ratio; PFS—progression free survival; CI—confidence interval; NR—not reported; Chemo—Chemotherapy.

**Table 3:** Trials of EGFR TKI’s in patients with advanced wild type NSCLC

Trial	Median Age (Years)	PS	EGFR status	Tx line	Regimen	Patients (n)	Median PFS, Months (95% CI)	HR for PFS (95% CI)
BR.21 [49]	62 (34–87)	0–3	EGFRmut (311/731) EGFRwt (132/731) Unknown (407/731)	≥2	Erlotinib Placebo	488 243	2.2 1.8 (95% CI NR)	0.61 (0.51–0.74)
TITAN [50]	59 (22–80)	0–2	EGFRmut (11/224) EGFRwt (149/224) Unknown (68/224)	2	Erlotinib Chemo *	203 221	6.3 weeks (6.1–6.9) 8.6 weeks (7.1–12.1)	1.19 (0.97–1.46)
TAILOR [51]	67 (35–83)	0–2	EFGRwt	2	Docetaxel Erlotinib	110 109	2.9 (2.4–3.8) 2.4 (2.1–2.6)	0.71 (0.53–0.95) *p* = 0.02
DELTA [52]	68 (37–82)	0–2	EGFRmut (51/301) EGFRwt (199/301) Other mut (5/301) Unknown (46/391)	≥2	Erlotinib Docetaxel	150 151	2 (1.3–2.8) 3.2 (2.8–4.0)	1.22 (0.97–1.55) *p* = 0.09
TOPICAL [5]	77 (72–82)	0–3	EGFRmut (28/670) EGFRwt (362/670) Unknown (280/670)	1	Erlotinib Placebo	350 320	2.8 (2.6–3) 2.6 (2.4–2.9)	0.83 (0.71–0.97) *p* = 0.019
Chen et al. [53]	77 (70–90)	0–3	EGFRmut (24/113) EGFRwt (36/113) Unknown (53/113)	1	Erlotinib Vin	57 56	4.6 2.5 (95% CI NR)	0.64 (0.43–0.96) *p* = 0.03
POLARSTAR [54]	<75: 7848/9907 75–84: 1911/9907 ≥85: 148/9907	0–4	NR	0 to ≥2	Erlotinib	9651	<75: 65 days (62–68) 75–84: 74 days (69–82) ≥85: 62 days (56–93)	*p* = 0.001
Jackman et al. [55]	75 (70–91)	0–2	EGFRmut (9/80) EGFRwt (34/80) Unknown (37/80)	1	Erlotinib	80	TTP: 3.5 (2.0–5.5)	--
IFCT-0301 [56]	Younger: 62 (30–69) Elderly: 73 (70–80)	2–3	Unknown (127/127)	1	Erlotinib Gem Docetaxel	43 42 42	Younger: 1.4 (1.1–1.9) Elderly: 2.3 (2.1–2.9)	0.57 (0.38–0.85) *p* = 0.004
SATURN [57]	60 (30–83)	0–1	EGFRmut (621/889) EGFRwt (121/889) Indeterminate (40/889) Unknown (107/889)	Maintenance	Erlotinib Placebo	438 451	12.3 11.1 (95% CI NR)	0.71 (0.62–0.82) *p* < 0.0001
INTEREST [58]	61 (27–84)	2–4	EGFRmut (44/1433) EGFRwt (253/1433) Unknown (1136/1433)	0–2	Gefitinib Docetaxel	723 710	2.2 2.7 (95% CI NR)	1.04 (0.93–1.18) *p* = 0.47
CTONG0806 [59]	57 (27–78)	0–1	EGFRwt 157/157	2	Pem Gefitinib	76 81	4.8 1.6 (95% CI NR)	0.54 (0.40–0.75) *p* < 0.0001
TIMELY [9]	36–90	0–3	Confirmed or suspected EGFRmut	1	Afatinib	39	7.9 (4.6–10.2)	NR
Ahn et al. [60]	NR	NR	EGFRwt (42/42)	3	Afatinib	38	4.1 weeks (3.9–8.0)	NR

* Chemotherapy—Pemetrexed or docetaxel (at investigator’s discretion). **Abbreviations**: PS—performance status; EGFR—epidermal growth factor receptor; Tx line—treatment line; HR—hazard ratio; PFS—progression free survival; CI—confidence interval; NR—not reported; Chemo—Chemotherapy, EGFRmut = EGFR mutation, EGFRwt = EGFR wild type, mut = mutation; TTP = time to progression; Vin-vinorelbine; Gem-gemcitabine; Pem-pemetrexed

**Table 4:** Trials of 1st line chemotherapy in elderly/PS2 patients with NSCLC.

Trial	Age; Years(Median)	PS	Regimen	Patients (n)	Median OS, Months (95% CI)	HR for OS (95% CI)	Median PFS, Months (95% CI)	HR for PFS (95% CI)	ORR (%)
MILES3/MILES4 [70]	75	0–2	Gem/Pem Cis + gem/Pem	268 263	7.5 9.6	HR 0.86, (0.70–1.04, *p* = 0.14)	3 4.6	HR 0.76, (0.63–0.92, *p* = 0.005)	8.5 15.5
Socinski et al. [71]	<70	0–2	Carbo + *nab*-PC Carbo + *sb*-PC	447 449	8.0 6.8	HR 0.999	6.0 5.8	HR 0.903	NR
	≥70	0–2	Carbo + *nab*-PC Carbo + *sb*-PC	74 82	19.9 10.4	HR 0.583	11.4 11.3	HR 0.687	NR
ABOUND 70+ [72]	7675	0–1	Carbo + *nab-*PC (weekly) Carbo + *nab*-PC (3 weekly)	71 72	15.2 16.2	HR 0.76 (0.46–1.26, *p* = 0.292)	3.9 7.0	HR 0.49 (0.30–0.79; *p* = 0.003)	23.9 40.3
ABOUND-PS2 [73]	67.5	2	Carbo + *nab*-PC +/− *nab*-PC maint	40	8.6 (5.1–13.2)	NR	4.4 (3.2–5.7)	NR	27.5
PARAMOUNT [74]	<70	0–1	Cis/Pem + maint Pem Cis/Pem	447	NR	0.75	NR	0.69 (0.54–0.90)	NR
	≥70	0–1	Cis/Pem + maint Pem Cis/Pem	92	NR	0.81	NR	0.35 (0.20–0.63)	NR

**Abbreviations:** PS—performance status; HR—hazard ratio; OS—overall survival; PFS—progression free survival; CI—confidence interval; ORR—objective response rate; NR—not reported; Gem—gemcitabine; Pem—pemetrexed; Cis—cisplatin; Carbo—carboplatin; nab-PC—nab-paclitaxel; sb-PC—solvent-based-paclitaxel; maint—maintenance.

**Table 5:** Subgroup analyses of elderly patients in trials of immune-checkpoint inhibitors in NSCLC.

Trial	Age;Years	Tx Line	PS	Regimen	Patients (n)	Median OS, Months (95% CI)	HR for OS (95% CI)	HR for PFS (95% CI)	TRAE (Grades 3–4), n (%)
KEYNOTE-024 [102]	<65	1	0–1	Pembro SOC	141	NR	NR	0.61 (0.40–0.92)	NR
	≥65	1	0–1	Pembro SOC	164	NR	NR	0.45 (0.29–0.70)	NR
KEYNOTE-189 [103]	<65	1	0–1	SOC + Pembro SOC + Placebo	312	NR	0.43 (0.31–0.61)	0.43 (0.32–0.56)	NR
	≥65	1	0–1	SOC + Pembro SOC + Placebo	304	NR	0.64 (0.43–0.95)	0.75 (0.55–1.02)	NR
CHECKMATE-227 [104]	<65	1	0–1	Nivo + Ipi Chemo *	156	NR	NR	0.51 (0.34−0.77)	NR
	≥65	1	0–1	Nivo + Ipi Chemo *	143	NR	NR	0.62 (0.40−0.97)	NR
	≥75	1	0–1	Nivo + Ipi Chemo *	27	NR	NR	0.42 (0.14−1.3)	NR
CHECKMATE- 153 [109]	<70	≥2	0–2	Nivo (to PD **) Nivo (1 year)	830	9.4 (8.3–10.9)	NR	NR	90 (11)
	≥70	≥2	0–2	Nivo (to PD **) Nivo (1 year)	544	10.3 (8.3–11.6)	NR	NR	73 (13)
CHECKMATE-171 [110] *Single arm, Phase II*	66 (31–86)	≥2	0–2	Nivo	809 (All pts)	9.9 (8.7–13.1)	NR	NR	95 (12)
	≥70	≥2	0–2	Nivo	279	11.2 (7.6–NR)	NR	NR	38 (14)

**Abbreviations:** PS—performance status; Tx line—treatment line; NR—not reported; HR—hazard ratio; OS—overall survival; PFS—progression free survival; CI—confidence interval; Pembro—Pembrolizumab; SOC—standard of care; Nivo—Nivolumab; Ipi—Ipilimumab; * Chemo—Chemotherapy (according to histological type); ** treatment to PD, unacceptable toxicity or withdrawal of consent.

**Table 6:** Subgroup analyses of patients with ECOG PS 2 in trials of immune-checkpoint inhibitors in previously treated NSCLC.

Trial	Age, Years Median (Range)	Tx Line	PS	Regimen	Patients (n)	Median OS, Months (95% CI)	TRAE Rate (All Grades), n (%)	TRAE Rate (Grades 3–4), n (%)
CHECKMATE-153 [109]	67 (29–93)	≥2	0–1	Nivo (to PD **) Nivo (1 year)	1230	10.5 (9.3–11.4)	766 (62)	146 (12)
	69 (45–91)	≥2	2	Nivo (to PD **) Nivo (1 year)	123	3.9 (3.1–6.3)	58 (47)	13 (11)
CHECKMATE-171 [110] *Single arm, Phase II*	66 (31–86)	≥2	0–2	Nivo	809 (All pts)	9.9 (8.7–13.1)	503 (50)	95 (12)
	68 (42–86)	≥2	2	Nivo	98	5.4 (3.9–8.3)	45 (46)	6 (6)

**Abbreviations:** PS—performance status; Tx line—treatment line; OS—overall survival; CI—confidence interval; TRAE—treatment-related adverse event; Nivo—Nivolumab; PD—progressive disease; pts—patients; ** treatment to PD, unacceptable toxicity or withdrawal of consent.
